# Phase Equilibria of Si-C-Cu System at 700 °C and 810 °C and Implications for Composite Processing

**DOI:** 10.3390/ma18153689

**Published:** 2025-08-06

**Authors:** Kun Liu, Zhenxiang Wu, Dong Luo, Xiaozhong Huang, Wei Yang, Peisheng Wang

**Affiliations:** 1 Hunan Key Laboratory of Advanced Fibers and Composites, Central South University, Changsha 410083, China; 2 Key Laboratory of Lithium Battery New Energy Materials and Devices of Jiangxi Education Department, Yichun University, Yichun 336000, China; 3 Hunan Boxiang New Materials Ltd., Changsha 410221, China; 4 Powder Metallurgy Research Institute, Central South University, Changsha 410083, China

**Keywords:** phase equilibria, isothermal section, Si-C-Cu

## Abstract

The phase equilibria of the Si-C-Cu ternary system at 700 °C and 810 °C were experimentally investigated for the first time. Fifteen key alloys were prepared via powder metallurgy and analyzed using X-ray diffraction (XRD), scanning electron microscopy (SEM), and electron probe microanalysis (EPMA). Isothermal sections were constructed based on the identified equilibrium phases. At 700 °C, eight single-phase regions and six three-phase regions—(C)+(Cu)+hcp, (C)+hcp+γ-Cu_33_Si_7_, (C)+γ-Cu_33_Si_7_+SiC, γ-Cu_33_Si_7_+SiC+ε-Cu_15_Si_4_, SiC+ε-Cu_15_Si_4_+η-Cu_3_Si(ht), and SiC+(Si)+η-Cu_3_Si(ht)—were determined. At 810 °C, nine single-phase regions and seven three-phase regions were identified. The solubility of C and Si/Cu in the various phases was quantified and found to be significantly higher at 810 °C compared to 700 °C. Key differences include the presence of the bcc (β) and liquid phases at 810 °C. The results demonstrate that higher temperatures promote increased mutual solubility and reaction tendencies among Cu, C, and Si. Motivated by these findings, the influence of vacuum hot pressing parameters on SiC-fiber-reinforced Cu composites (SiC_f_/Cu) was investigated. The optimal processing condition (1050 °C, 60 MPa, 90 min) yielded a high bending strength of 998.61 MPa, attributed to enhanced diffusion and interfacial bonding facilitated by the high-temperature phase equilibria. This work provides essential fundamental data for understanding interactions and guiding processing in SiC-reinforced Cu composites.

## 1. Introduction

Copper has attracted considerable attention and has been widely used because of its low cost, excellent electrical and thermal conductivity, strong corrosion resistance, and potential for biomedical applications [1,2,3]. However, the mechanical properties of pure copper are inadequate for service conditions involving high-stress or high-speed operation [4]. In contrast, silicon-carbide-fiber-reinforced Cu composites (SiC_f_/Cu) have excellent thermophysical properties, such as high conductivity, a low thermal expansion coefficient, excellent mechanical properties, and wear resistance [5,6,7,8]. These characteristics make SiC_f_/Cu composites highly promising for demanding applications such as heat dissipation in electronic packaging and plasma-facing components in nuclear fusion reactors [9,10,11].

Phase diagrams are essential tools for guiding the compositional design and processing optimization of composites [12,13]. To improve the performance and reliability of SiC_f_/Cu composites, it is necessary to accurately understand the phase diagram of the Si-C-Cu ternary system. Such knowledge is particularly vital for predicting and controlling interfacial reactions and diffusion processes during composite fabrication at elevated temperatures. However, investigating this system presents significant challenges due to the extremely high melting points and low volatility of SiC and C, making conventional melting techniques unsuitable. Consequently, although the constituent binary systems (Cu-C, C-Si, Cu-Si) have been studied, the ternary Si-C-Cu phase diagram remains experimentally unexplored.

The solubility of C in liquid Cu was reported by López [14] and Oden [15]. Predel [16] presented a Cu-C phase diagram. Subsequently, Franke [17] performed a detailed evaluation of the system, summarizing the structure of the phases and the invariant reactions. However, the thermodynamic parameters were not included in the evaluation by Franke [17]. Chen [18] thermodynamically optimized the Cu-C system using the data from the above literature, and the calculated Cu-C phase diagram is shown in the Supplementary Materials.

The thermodynamic evaluation of the C-Si system by Gröbner et al. [19] agrees well with the available literature data. The calculated phase diagram obtained according to Gröbner’s optimized parameters is shown in the Supplementary Materials.

Following the evaluations of Olesinski et al. [20] in 1986 and Yan [21] in 2000, the Cu-Si system saw limited new experimental data until Hallstedt et al. [22] performed a comprehensive re-assessment using the CALPHAD method. This optimized data is adopted in this paper. As shown in the Supplementary Materials, their optimized phase diagram agrees well with experimental data.

The temperatures of 700 °C and 810 °C were selected as key points for constructing the isothermal sections of the Si-C-Cu system, based on the distinct phase transformations observed in the Cu-Si binary system. At 700 °C, (Cu), the hexagonal close-packed (hcp) phase, γ-Cu_33_Si_7_, ε-Cu_15_Si_4_, η-Cu_3_Si (ht), and the Si phase are all thermodynamically stable. By 810 °C, a significant phase evolution occurs: the ε-Cu_15_Si_4_ phase disappears, and a body-centered cubic (bcc) phase emerges, along with the formation of a liquid phase. The stable phases at this temperature include the Cu phase, hcp phase, bcc phase, δ-Cu_33_Si_7_, η-Cu_3_Si (ht), liquid phase, and Si phase. Notably, 740 °C represents the critical transformation temperature from γ-Cu_33_Si_7_ to δ-Cu_33_Si_7_. Between 700 °C and 810 °C, the disappearance of the ε-Cu_15_Si_4_ phase, the emergence of the bcc and liquid phases, and structural transitions in Cu-Si intermetallic compounds collectively indicate a key temperature range for phase evolution. The selection of 700 °C and 810 °C allows for the coverage of this critical transformation interval, enabling a systematic comparison of phase stability and transformation behavior in the Si-C-Cu system. This temperature pair provides valuable insights into the phase equilibria and thermodynamic basis of the system, which is of significant relevance for the processing of Cu-based composites.

Accordingly, this research aims to experimentally establish the isothermal sections of the Si-C-Cu system at 700 °C and 810 °C for the first time, utilizing powder metallurgy techniques. Furthermore, leveraging the insights gained into temperature-dependent solubility and reactivity, the influence of key processing parameters on the microstructure and mechanical properties of SiC_f_/Cu composites is investigated, demonstrating the practical application of the fundamental phase diagram data for composite optimization.

## 2. Experimental Procedure

Considering the absence of a liquid phase during carbon sublimation under atmospheric pressure, arc melting is deemed an unsuitable method for sample preparation in the Si-C-Cu system. Therefore, powder metallurgy was employed to fabricate the ternary Si-C-Cu samples. Fifteen key ternary samples were designed based on thermodynamic predictions from the binary subsystems of Si-C-Cu. Six compositions with 5 at.% C were selected to examine the isothermal section at 700 °C, where carbon solubility is limited and its impact on phase formation is minimal. Five compositions with 20 at.% C were chosen for the 810 °C isothermal section to explore the effects of higher carbon content, which promotes the formation of additional phases and potentially liquid phases due to the increased solubility and reactivity at this temperature.

The crystallographic characteristics of the phases within the Si-C-Cu system are summarized in Table 1, and the nominal compositions of the as-prepared samples are presented in Table 2.

High-purity copper (99.99%), graphite (99.99%), and silicon (99.99%) powders with a particle size of 325 mesh (Zhongnuo New Materials (Beijing) Technology Co., Ltd., Beijing, China) were selected as raw materials. The powders were proportionally weighed using an analytical balance, homogenized in a mixer for 180 min, and subsequently consolidated via cold uniaxial pressing using a 30T hydraulic press (Tengzhou Tengke Hydraulic Equipment Co., Ltd., Tengzhou, China) at a pressure of 200 MPa for a duration of 8 s. Subsequently, sintering was carried out under argon protection at a suitable temperature for 3 h. The specific sintering temperatures are shown in Table 2. To promote phase equilibrium, the samples were cooled with the furnace, sealed in quartz tubes under argon, annealed at 700 °C or 810 °C for 30 days, and finally quenched in water to preserve the high-temperature phase structure. The samples were cut into small pieces and examined by XRD, SEM, and EPMA, respectively.

X-ray diffraction measurements were performed using a Rigaku Smartlab SE diffractometer (Tokyo, Japan) equipped with a Cu target (λ = 1.541862 Å). The operating conditions included an accelerating voltage of 40 kV and a current of 40 mA. Microstructural observations were conducted using a TESCAN MIRA4 LMH scanning electron microscope (Brno, Czech Republic). Energy-dispersive spectroscopy (EDS) was carried out with an Oxford Instruments Ultim Max 40 detector (Abingdon, UK). The SEM was operated at an accelerating voltage of 15 kV, under vacuum conditions below 10^−3^ Pa, and with a resolution of 1.4 nm. Elemental analysis was performed using a JEOL JXA-8530F electron probe microanalyzer (Tokyo, Japan). The instrument was operated at an accelerating voltage of 15 kV under vacuum conditions below 10^−3^ Pa, with a resolution of 3 nm.

To investigate the application of phase equilibria insights, SiC-fiber-reinforced copper matrix composites were fabricated via vacuum hot pressing. SiC fibers (Zeralon 201, Hunan Zerafber New Materials Co., Ltd., Changsha, China) were arranged within a copper matrix. Nine composite samples (designated Cu-1 to Cu-9) were prepared under different combinations of temperature (950 °C, 1000 °C, 1050 °C), pressure (40 MPa, 50 MPa, 60 MPa), and holding time at temperature and pressure (60 min, 90 min, 120 min), as detailed in Table 3. After processing, the composites were machined into bend test specimens with specified dimensions (thickness and width listed in Table 3). Three-point bending tests were conducted to determine the bending strength, with the maximum load recorded and strength calculated (Table 3). Cross-sectional metallographic analysis was performed on selected samples (Cu-1, Cu-2, Cu-5) to examine fiber distribution, matrix infiltration, porosity, and fiber–matrix interfacial bonding using SEM and EPMA.

## 3. Results and Discussions

### 3.1. Isothermal Sections of Si-C-Cu at 700 °C

**Figure 1:**
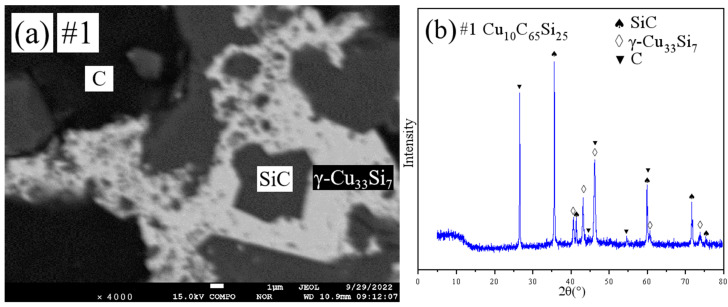
SEM and XRD patterns of sample 1: (**a**) SEM; (**b**) XRD.

**Figure 2:**
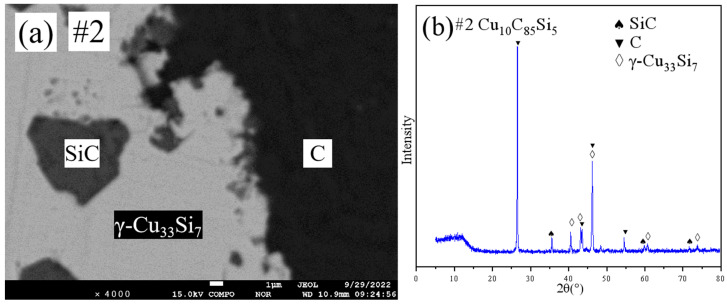
SEM and XRD patterns of sample 2: (**a**) SEM; (**b**) XRD.

**Figure 3:**
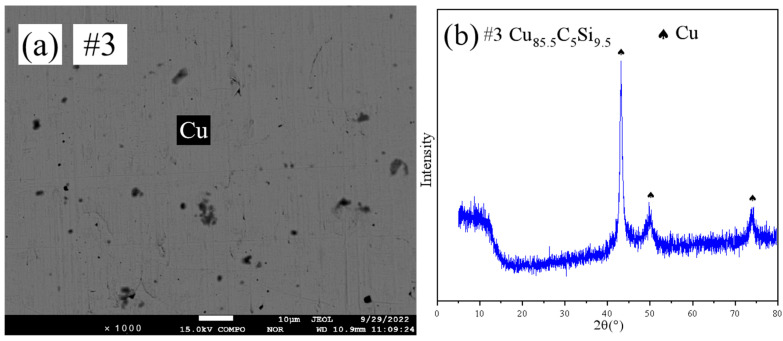
SEM and XRD patterns of sample 3: (**a**) SEM; (**b**) XRD.

**Figure 4:**
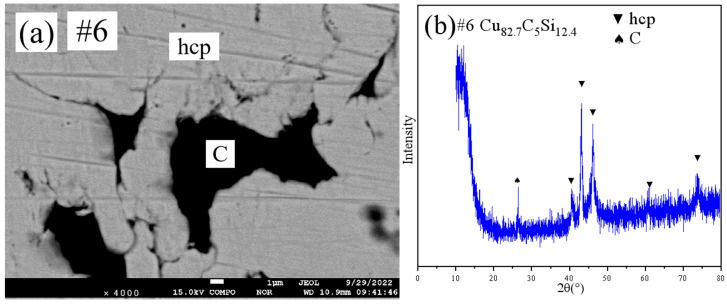
SEM and XRD patterns of sample 6: (**a**) SEM; (**b**) XRD.

**Figure 5:**
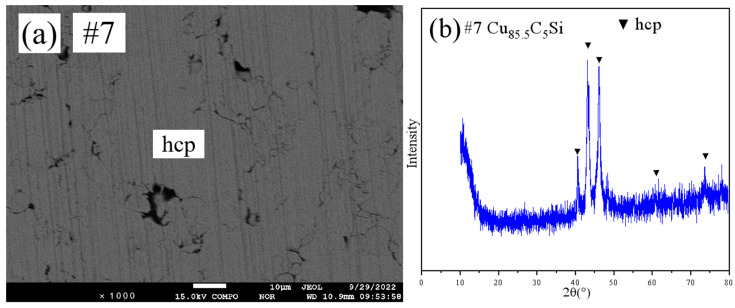
SEM and XRD patterns of sample 7: (**a**) SEM; (**b**) XRD.

**Figure 6:**
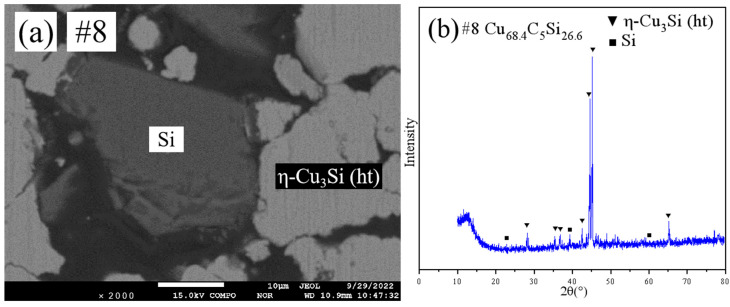
SEM and XRD patterns of sample 8: (**a**) SEM; (**b**) XRD.

The equilibrium phases identified in the fifteen annealed samples, determined through combined XRD, SEM, and EPMA analysis, are listed in Table 2. Representative microstructural and diffraction data are presented in Figure 1, Figure 2, Figure 3, Figure 4, Figure 5 and Figure 6.

SEM analysis (Figure 1a and Figure 2a) revealed three-phase microstructures. Combined XRD (Figure 1b and Figure 2b) and EPMA quantification confirmed that the two samples reside within the γ-Cu_33_Si_7_+SiC+(C) triple-phase region.

Figure 3 presents the SEM micrographs and XRD patterns of sample #3. The results from XRD and EPMA analyses confirm that sample #3 is in the (Cu) solid solution single-phase region. For samples #4 and #5, their SEM micrographs and XRD patterns are provided in the Supplementary Materials. Similarly, the XRD and EPMA analyses indicate that both samples #4 and #5 also reside within the (Cu) solid solution single-phase region, consistent with the findings for sample #3.

Figure 4 shows the SEM pattern and XRD pattern of sample #6. SEM (Figure 4a) indicated a two-phase structure. XRD (Figure 4b) and EPMA identified the equilibrium phases as within the hcp+(C) two-phase region.

Figure 5 shows the SEM pattern and XRD pattern of sample #7. SEM (Figure 5a) showed a single-phase microstructure. XRD (Figure 5b) and EPMA confirmed that this sample lay within the hcp single-phase region.

Figure 6 shows the SEM pattern and XRD pattern of sample #8. SEM (Figure 6a) revealed a two-phase structure. XRD (Figure 6b) and EPMA identified the phases as within the η-Cu_3_Si(ht)+Si two-phase region.

**Figure 7:**
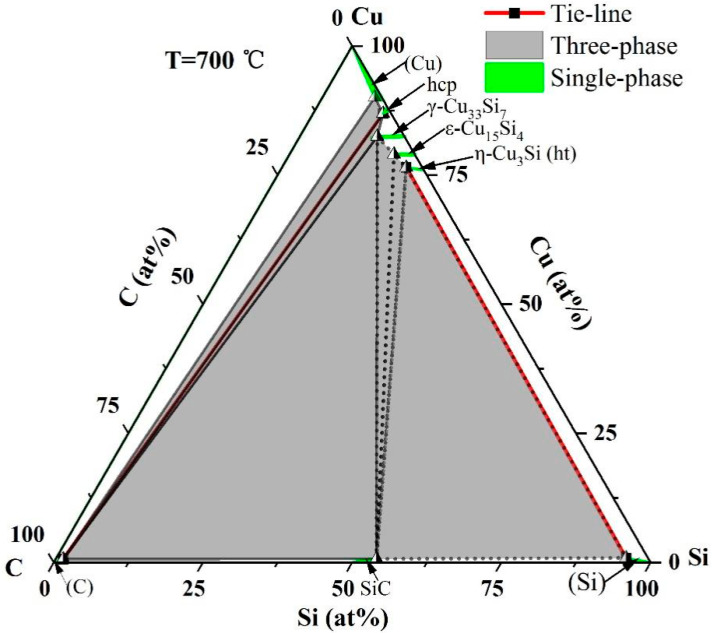
Isothermal section of Si-C-Cu at 700 °C.

The isothermal section of the Si-C-Cu system at 700 °C, derived from the experimental data, is provided in Figure 7. In the figure, solid lines delineate regions that have been experimentally confirmed, while dashed lines indicate boundaries that are inferred based on thermodynamic predictions and phase equilibrium relationships rather than directly verified by experimental samples. There are, in total, eight single-phase regions and six three-phase regions. Among them, (Cu), hcp, γ-Cu_33_Si_7_, ε-Cu_15_Si_4_, η-Cu_3_Si(ht), (Si), (C), and SiC are single-phase regions, indicated in green on the diagram; (C)+(Cu)+hcp, (C)+hcp+γ-Cu_33_Si_7_, (C)+γ-Cu_33_Si_7_+SiC, γ-Cu_33_Si_7_+SiC+ε-Cu_15_Si_4_, SiC+ε-Cu_15_Si_4_+η-Cu_3_Si(ht), and SiC+(Si)+η-Cu_3_Si(ht) are three-phase areas, indicated in gray on the diagram; and the other white areas are two-phase areas. Each single phase has a certain solid solubility. The solubilities of Si and C in the (Cu) phase are 10.6 at.% and 1.2 at.%, respectively. The solubility of carbon in the (Si) phase reaches 3.7 at.%, with a copper solubility of 0.9 at.%. In the (C) phase, silicon and copper exhibit solubilities of 1.3 at.% and 0.7 at.%, respectively. For the hcp phase, the solubility of carbon is 1.4 at.%. Among the intermetallic phases, the solubility of C is measured as 4.6 at.% in γ-Cu_33_Si_7_, 3.5 at.% in ε-Cu_15_Si_4_, and 2.7 at.% in η-Cu_3_Si(ht). Additionally, the SiC phase accommodates up to 0.7 at.% Cu. From the 700 °C isothermal section, it can also be seen that when the Cu content is greater than 85.6 at.%, a small amount of Si will preferentially react with Cu to generate copper silicon compounds, rather than reacting with C to generate SiC; as the Cu content decreases and the Si content increases, Si will react with C to form SiC.

### 3.2. Isothermal Sections of Si-C-Cu at 810 °C

Analysis of samples annealed at 810 °C (Table 2, Figure 8, Figure 9, Figure 10, Figure 11, Figure 12, Figure 13 and Figure 14) led to the construction of the isothermal section shown in Figure 15.

**Figure 8:**
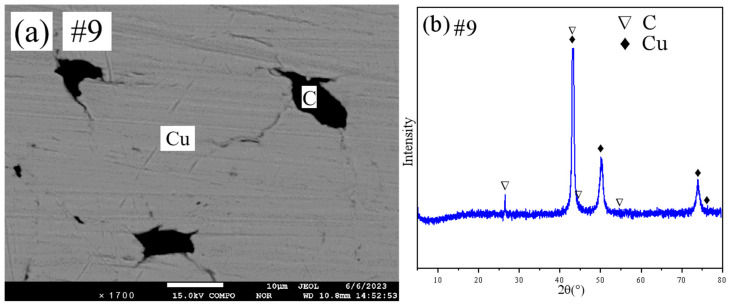
SEM and XRD patterns of sample 9: (**a**) SEM; (**b**) XRD.

Figure 8a,b show the SEM pattern and XRD pattern of sample #9, respectively. From the SEM pattern analysis, it can be obtained that sample #9 is in the two-phase region. Combined with the quantitative results of XRD and EPMA, it can be determined that sample #9 is in the (Cu)+(C) two-phase region.

**Figure 9:**
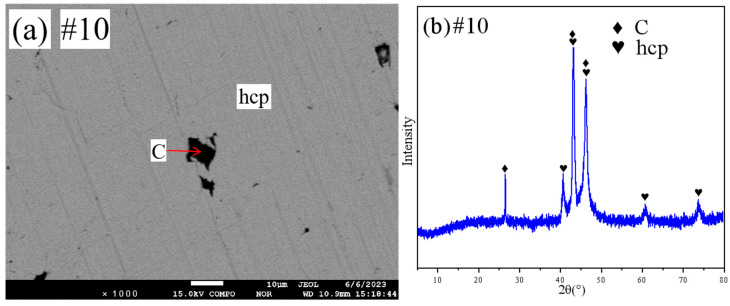
SEM and XRD patterns of sample 10: (**a**) SEM; (**b**) XRD.

The SEM patterns and XRD patterns of sample #10 are shown in Figure 9a,b. From the SEM pattern analysis, it can be obtained that sample #10 is in the two-phase region. Combined with the quantitative results of XRD and EPMA, it can be determined that sample #10 is in the hcp+ (C) two-phase region.

**Figure 10:**
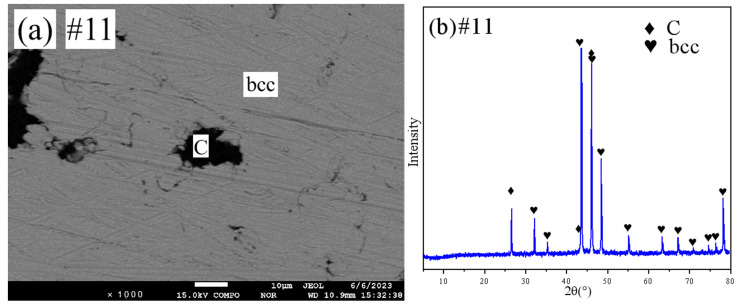
SEM and XRD patterns of sample 11: (**a**) SEM; (**b**) XRD.

**Figure 11:**
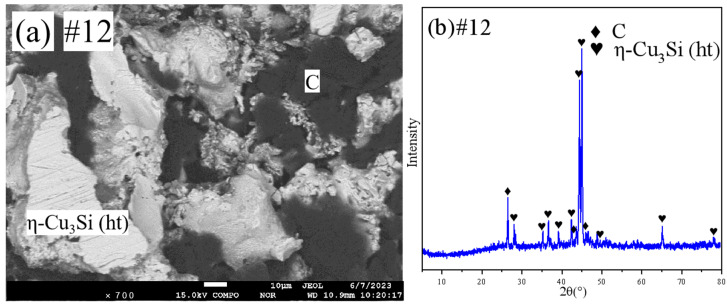
SEM and XRD patterns of sample 12: (**a**) SEM; (**b**) XRD.

Figure 10 shows the SEM and XRD patterns of sample 11. Figure 11 shows the SEM and XRD patterns of sample 12. They are all in the two-phase region. Combined with the EPMA results, it is clear that sample 11 is in the (C)+bcc two-phase region and sample 12 is in the (C)+η-Cu_3_Si(ht) two-phase region.

**Figure 12:**
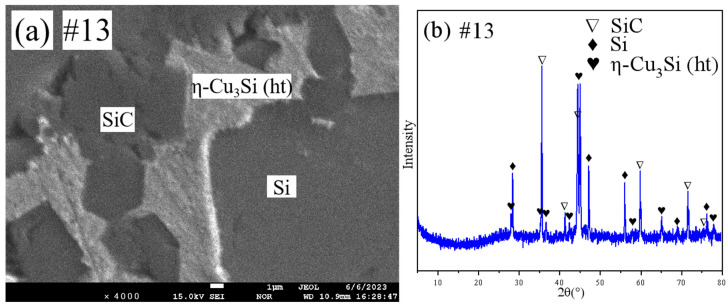
SEM and XRD patterns of sample 13: (**a**) SEM; (**b**) XRD.

**Figure 13:**
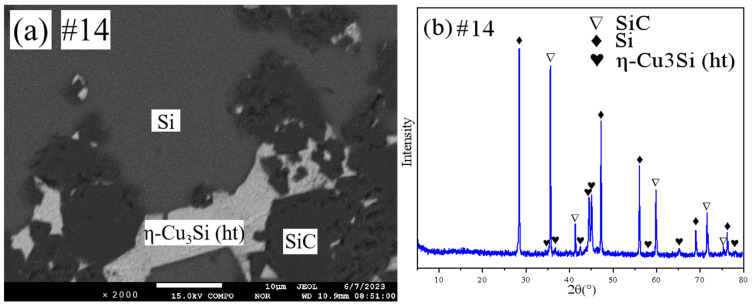
SEM and XRD patterns of sample 14: (**a**) SEM; (**b**) XRD.

Representative SEM micrographs and corresponding XRD patterns of samples 13 and 14 are shown in Figure 12 and Figure 13, respectively. Both samples are in the three-phase region. Combined with the results of EPMA, it is clear that both sample 13 and sample 14 are in the SiC+(Si)+η-Cu_3_Si(ht) three-phase region.

**Figure 14:**
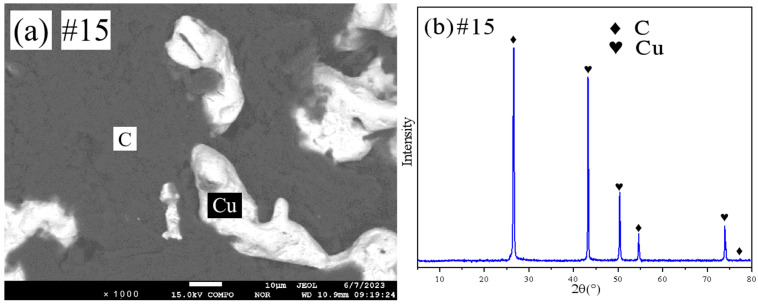
SEM and XRD patterns of sample 15: (**a**) SEM; (**b**) XRD.

Figure 14 provides the XRD and SEM data for sample 15. Based on the analysis of this figure, sample 15 is identified as being within the (Cu)+(C) two-phase region.

**Figure 15:**
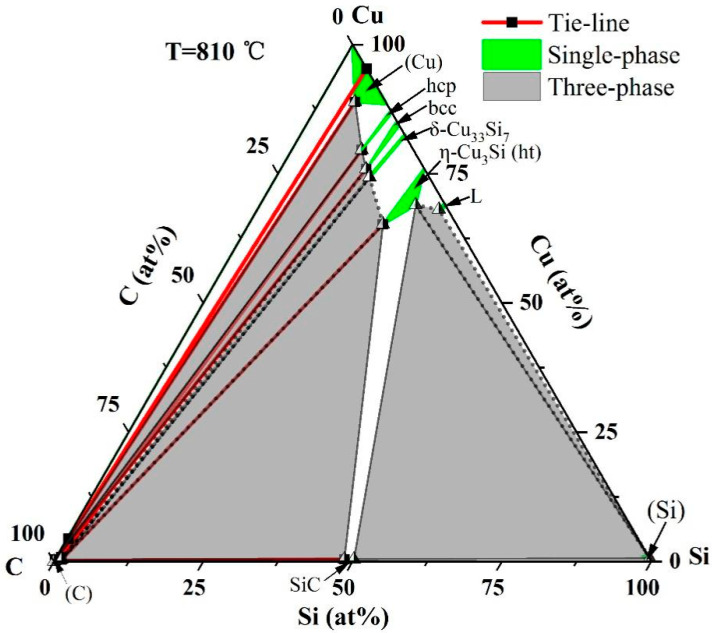
Isothermal section of Si-C-Cu at 810 °C.

Utilizing the experimental data, the isothermal section of the Si-C-Cu system at 810 °C was established and is depicted in Figure 15. In the figure, solid lines represent phase regions that have been experimentally confirmed based on the data, while dashed lines indicate phase boundaries inferred from thermodynamic predictions, as these regions were not directly confirmed by experimental samples. As shown in Figure 15, in the isothermal section at 810 °C, there are a total of nine single-phase regions and seven three-phase regions, with the remaining being two-phase regions. Among them, (Cu), hcp, bcc, δ-Cu_33_Si_7_, η-Cu_3_Si(ht), (Si), (C), SiC, and liquid are single-phase regions, indicated in green on the diagram; (C)+(Cu)+hcp, (C)+hcp+bcc, (C)+δ-Cu_33_Si_7_+bcc, (C)+δ-Cu_33_Si_7_+η-Cu_3_Si(ht), SiC+(C)+η-Cu_3_Si(ht), SiC+(Si)+η-Cu_3_Si(ht), and Liquid+(Si)+η-Cu_3_Si(ht) are three-phase areas, indicated in gray on the diagram; and the other white areas are two-phase areas. Each single phase has a certain solid solubility. The solubilities of Si and C in the (Cu) phase are 6.0 at.% and 5.1 at.%, respectively. In the (Si) phase, the solubility of Cu is 0.6 at.%. In the (C) phase, the solubility of Si is 1.1 at.% and the solubility of Cu is 0.4 at.%. In the hcp phase, the solubility of C is 8.7 at.%. In the bcc phase, the solubility of C is 9.8 at.%. In the δ-Cu_33_Si_7_ phase, the solubility of C is 10.0 at.%. In the η-Cu_3_Si(ht) phase, the solubility of C is 12.3 at.%. In the SiC phase, the solubility of Cu is 0.4 at.%. In the liquid phase, the solubility of C is 1.5 at.%. From the 810 °C isothermal section, it can also be seen that when the Cu content is greater than 68.9 at.%, a small amount of Si will preferentially react with Cu to generate copper silicon compounds, rather than react with C to generate SiC; as the Cu content decreases and the Si content increases, Si will react with C to form SiC.

### 3.3. Comparison of 700 °C and 810 °C Isothermal Sections

Significant differences exist between the phase equilibria at 700 °C and 800 °C. The solid solubility of C (and Si/Cu in specific phases) is substantially higher at 810 °C compared to 700 °C across nearly all phases, reflecting the expected increase in solubility with temperature. The bcc (β) phase and the liquid phase are stable at 810 °C but absent at 700 °C. The ε-Cu_15_Si_4_ phase is stable at 700 °C but absent at 810 °C.

### 3.4. Implications for SiC-Fiber-Reinforced Cu Composite Processing

Motivated by the observed significant increase in mutual solubility and reactivity between Cu, C, and Si with temperature in the Si-C-Cu isothermal sections (Section 3.1, Section 3.2 and Section 3.3),the influence of vacuum hot pressing parameters on the microstructure and mechanical properties of SiC-fiber-reinforced copper matrix composites (SiC_f_/Cu) was investigated.

The bending strength results for the nine composite samples (Cu-1 to Cu-9) processed under different conditions are summarized in Table 3. The sample fabricated under the Cu-9 condition (1050 °C, 60 MPa, holding for 90 min) exhibited the highest bending strength of 998.61 MPa, significantly outperforming samples processed at lower temperatures and/or pressures.

**Figure 16:**
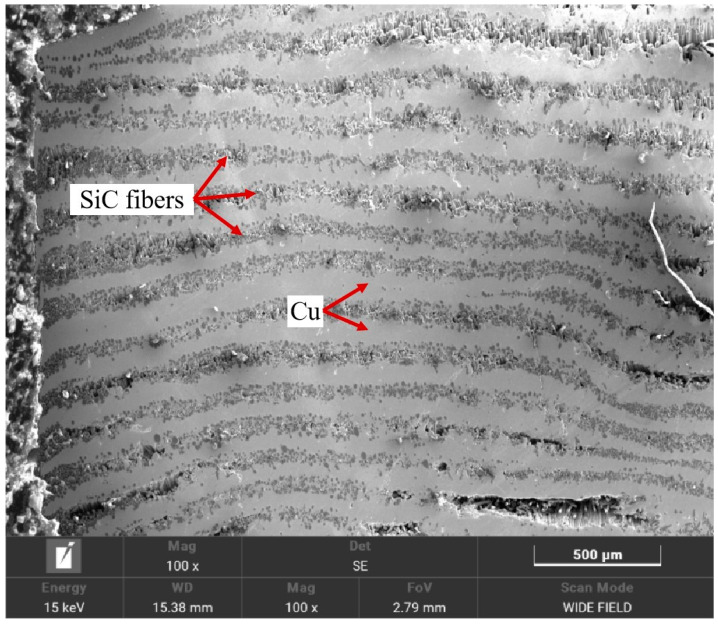
SEM image of cross-section of Cu-1.

**Figure 17:**
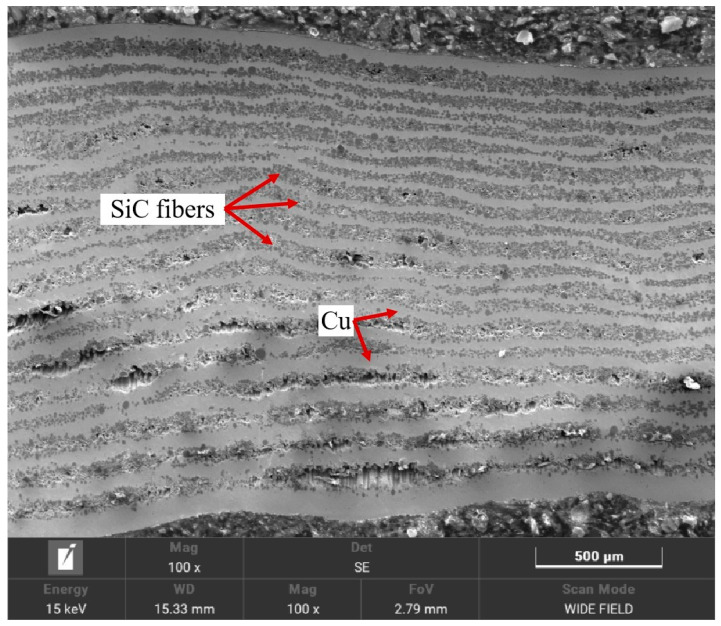
SEM image of cross-section of Cu-2.

**Figure 18:**
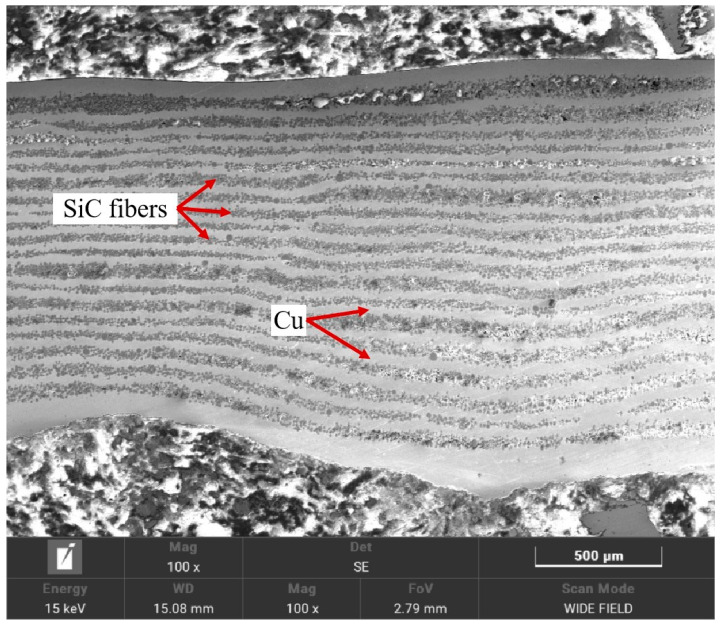
SEM image of cross-section of Cu-5.

To elucidate the mechanism behind this performance variation, SEM analyses were performed on representative samples: Cu-1 (low strength: 187 MPa, processed at 950 °C/40 MPa/60 min), Cu-2 (intermediate strength: 400 MPa, 1000 °C/40 MPa/90 min), and Cu-5 (higher strength: 713 MPa, 1000 °C/50 MPa/120 min). Their cross-sectional microstructures are shown in Figure 16, Figure 17 and Figure 18. The low-strength Cu-1 sample (Figure 16) exhibited numerous pores, voids, and poorly infiltrated regions where the Cu matrix failed to fully coat the SiC fibers. This lack of intimate contact prevents effective load transfer. The Cu-2 sample (Figure 17) showed improved infiltration but still contained noticeable porosity and some areas of poor fiber–matrix bonding. In contrast, the Cu-5 sample (Figure 18) displayed significantly fewer defects, with fibers uniformly distributed and tightly bonded to the Cu matrix, enabling effective reinforcement. The optimal processing condition (Sample Cu-9, 1050 °C, 60 MPa, 90 min) yielded a high bending strength of 998.61 MPa. At this temperature, the presence of intermetallic phases like η-Cu_3_Si and δ-Cu_33_Si_7_ contributed to enhanced diffusion and interfacial bonding between the SiC fibers and the Cu matrix. This improved interaction is attributed to the higher solubility and reactivity of Cu, C, and Si at elevated temperatures, facilitating better matrix infiltration and reduced porosity.

The experimental determination of the Si-C-Cu isothermal sections at 700 °C and 810 °C provides foundational data critical for understanding interactions in this system. The key findings—significantly increased solubility and reactivity with temperature, distinct phase stabilities, and the emergence of liquid phases—have direct implications for processing technologies involving these elements.

As demonstrated above, these phase equilibria insights offer a scientific basis for optimizing the fabrication of SiC-reinforced Cu composites. The superior performance achieved at 1050 °C and 60 MPa is attributed to the synergy of thermally activated processes, driven by higher solubilities (e.g., C in (Cu), Cu-Si phases) and reactivity, enhancing diffusion and interfacial bonding, and potential transient liquid-phase sintering/bonding facilitated by temperatures exceeding the Cu-Si eutectic (~802 °C), improving wettability and mass transport.

This work establishes a clear link between fundamental ternary phase equilibria and the practical optimization of composite manufacturing processes, highlighting the value of phase diagram knowledge in materials engineering.

## 4. Conclusions

1. This work presents the first experimental determination of the isothermal sections of the Si-C-Cu system at 700 °C and 810 °C using powder metallurgy, which serves as a fundamental reference for further studies on this ternary system.
2. In the Si-C-Cu system, the solubility of each single phase in the isothermal section at 810 °C is much larger than that of each phase in the isothermal cross-section at 700 °C.
3. Different phases appear in the 810 °C and 700 °C isothermal sections. Bcc and liquid phases appear in the 810 °C isothermal section, while the ε-Cu_15_Si_4_ phase appears in the 700 °C isothermal section.
4. As the temperature increases, Cu, C, and Si are more likely to react with each other, and the reaction area also increases.
5. The fundamental phase equilibria data, particularly the enhanced solubility and reactivity at higher temperatures, provide critical guidance for processing SiC-fiber-reinforced Cu composites. Vacuum hot pressing at 1050 °C and 60 MPa for 90 min was identified as the optimal condition, yielding the highest bending strength (998.61 MPa). This performance is attributed to improved matrix infiltration, reduced porosity, and enhanced fiber–matrix interfacial bonding, enabled by the high-temperature diffusion and reaction kinetics predicted by the phase diagram.

## Figures and Tables

**Table 1:** A summary of the crystallographic information for the phases of the Si-C-Cu system.

Phase Name	Crystallographic Information					
	Prototype	Pearson Symbol	Space Group	Lattice Parameters (nm)	Database	Ref.
(Cu), fcc, α	Cu	cF4	Fm-3m	a = 0.36149;	FCC_A1	[22,23]
hcp, κ	Mg	hP2	P6_3_/mmc	a = 0.25622; c = 0.41823;	HCP_A3	[22,23]
bcc, β	W	cI2	Im-3m	a = 0.2854;	BCC_A2	[22,23]
δ-Cu_33_Si_7_	-	hP *	P6_3_/mmc	a = 0.4036; c = 0.4943;	CU33SI7_DELTA	[22,23]
γ-Cu_33_Si_7_, Cu_56_Si_11_	β-Mn	cP20	P4_1_32	a = 0.6198;	CU33SI7_GAMA	[22,23]
ε-Cu_15_Si_4_	Cu_15_Si_4_	cI76	I-43d	a = 0.9718;	CU15SI4_ EPSILON	[22,23]
η-Cu_3_Si (ht), Cu_19_Si_6_	-	hR *	R-3m	a = 0.247;	CU3SI_HT	[22,23]
η′-Cu_3_Si (it)	η′-Cu_3_Si	hR9	R-3	a = 0.472;	CU3SI_MT	[22,23]
η′′-Cu_3_Si (lt)	-	oC *	?	a = 7.676; b = 0.7; c = 2.194;	CU3SI_LT	[22,23]
(Si)	C	cF8	Fd-3m	a = 0.54306;	DIAMOND_A4	[22,23]
SiC	ZnSc	cF8	F-43m	a = 0.43596;	SIC	[24]
(C)	C	hP4	P6_3_/mmc	a = 0.2461; c = 0.6704;	GRAPHITE	[24]

Note: The asterisk (*) denotes a variable or an uncertain quantity.

**Table 2:** Summary of EPMA for Si-C-Cu samples.

Sample No.	Nominal Composition (at.%)			Heat Treatment (°C)	Equilibrium Phase Composition (at.%)			Equilibrium Phase Constituent
	Si	Cu	C		Si	Cu	C	
1	25	15	65	1400-2 h/700-30 days	12.98	82.46	4.56	γ-Cu_33_Si_7_
					53.72	0.68	45.59	SiC
					4.35	4.18	91.47	(C)
2	5	10	85	1400-2 h/700-30 days	52.47	0.66	46.87	SiC
					12.14	82.27	5.59	γ-Cu_33_Si_7_
					0.20	0.248	99.55	(C)
3	9.5	85.5	5	1310-3 h/700-30 days	8.753	90.03	1.214	(Cu)
4	10.5	84.6	5	1020-3 h/700-30 days	9.85	88.88	1.26	(Cu)
5	11.4	83.6	5	1020-3 h/700-30 days	11.05	87.81	1.13	(Cu)
6	12.4	82.7	5	1050-3 h/700-30 days	11.73	86.90	1.37	hcp
					1.26	0.65	98.09	(C)
7	14.3	80.8	5	900-3 h/700-30 days	13.38	85.56	1.06	hcp
8	26.6	68.4	5	800-3 h/700-30 days	95.46	0.852	3.688	Si
					20.83	76.44	2.73	η-Cu_3_Si (ht)
9	5.0	75.0	20.0	900-3 h/810-30 days	6.0	88.9	5.1	(Cu)
					0.1	0.4	99.5	(C)
10	11.1	68.9	20.0	810-3 h/810-30 days	0.8	0.8	98.4	(C)
					11.7	79.6	8.7	hcp
11	13.3	66.7	20.0	810-3 h/810-30 days	0.1	0.4	99.5	(C)
					15.9	84.1	0.0	bcc
12	21.9	58.1	20.0	810-3 h/810-30 days	1.1	0.4	98.6	(C)
					22.5	65.2	12.3	η-Cu_3_Si (ht)
13	50.0	30.0	20.0	1400-3 h/810-30 days	50.0	0.4	49.6	SiC
					99.4	0.6	0.0	(Si)
					26.1	68.9	5.0	η-Cu_3_Si (ht)
14	65.0	10.0	25.0	1400-3 h/810-30 days	48.4	0.4	51.2	SiC
					22.5	65.2	12.3	η-Cu_3_Si (ht)
					99.6	0.4	0.0	(Si)
15	1.0	19.0	80.0	1400-3 h/810-30 days	4.7	95.3	0.0	(Cu)
					0.0	0.2	99.7	(C)

**Table 3:** Mechanical properties of nine samples prepared under different conditions.

Sample No.	Temperature/°C	Pressure/MPa	Holding Time/min	Sample Thickness/mm	Sample Width/mm	Load/N	Bending Strength/MPa
Cu-1	950	40	60	1.48	10.10	91.94	187
Cu-2	1000	40	90	1.18	10.12	125.3	400.15
Cu-3	1050	40	120	1.16	10.11	205.83	680.87
Cu-4	950	50	90	1.47	10.11	118.38	243.84
Cu-5	1000	50	120	1.10	10.13	194.23	713.06
Cu-6	1050	50	60	0.98	10.11	183.73	758.83
Cu-7	950	60	120	1.09	10.18	188.90	702.83
Cu-8	1000	60	60	1.01	10.19	174.85	756.92
Cu-9	1050	60	90	0.87	10.10	155.80	917.08
Cu-9	1050	60	90	0.89	10.21	179.47	998.61

## Data Availability

The original contributions presented in this study are included in the article/Supplementary Materials. Further inquiries can be directed to the corresponding authors.

## Supplementary Materials

The following supporting information can be downloaded at: https://www.mdpi.com/article/10.3390/ma18153689/s1, Figure S1: Cu-C phase diagram obtained from the results of Chen [18]; Figure S2: C-Si phase diagram obtained from the results of Gröbner [19]; Figure S3: Cu-Si phase diagram obtained from the results of Hallstedt [22]; Figure S4. SEM and XRD patterns of sample 4: (a) SEM; (b) XRD; Figure S5. SEM and XRD patterns of sample 5: (a) SEM; (b) XRD.
