# Supplementary material for: Phase Equilibria of Si-C-Cu System at 700 °C and 810 °C and Implications for Composite Processing
Source: Materials (Basel). 2025 Aug 6;18(15):3689. doi: 10.3390/ma18153689 (PMC12348228; doi:10.3390/ma18153689)
Supplement: Supplementary file 1 [file materials-18-03689-s001.zip › materials-3765853-supplementary.pdf]

Supplementary Materials

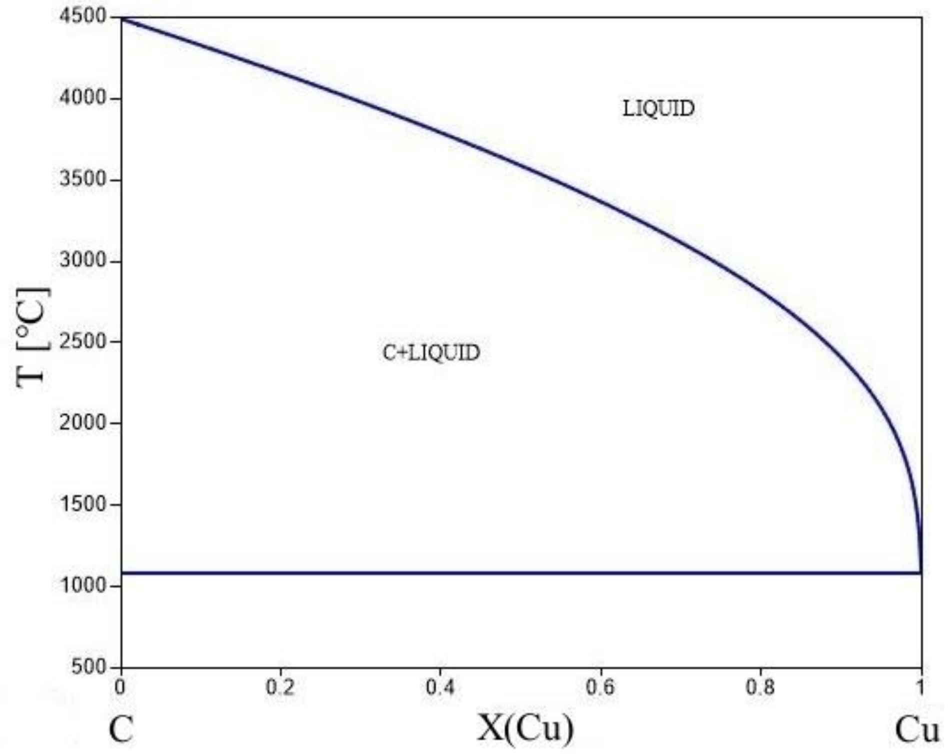

Figure S1. Cu-C phase diagram obtained from the results of Chen [18].

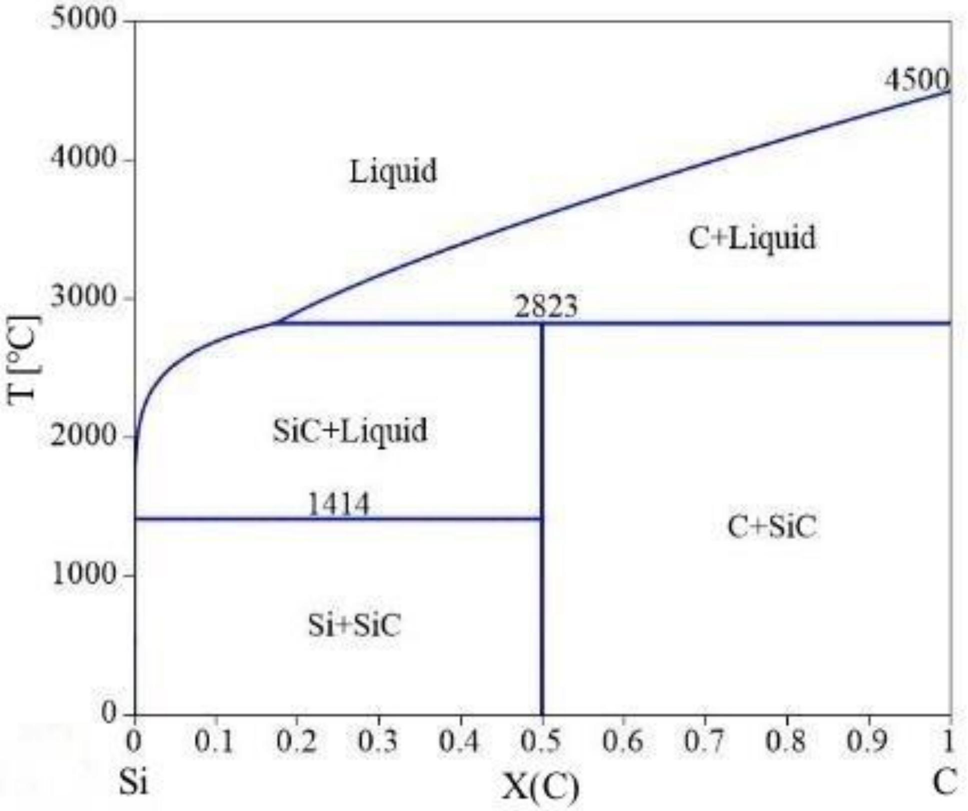

Figure S2. C-Si phase diagram obtained from the results of Gröbner [19].

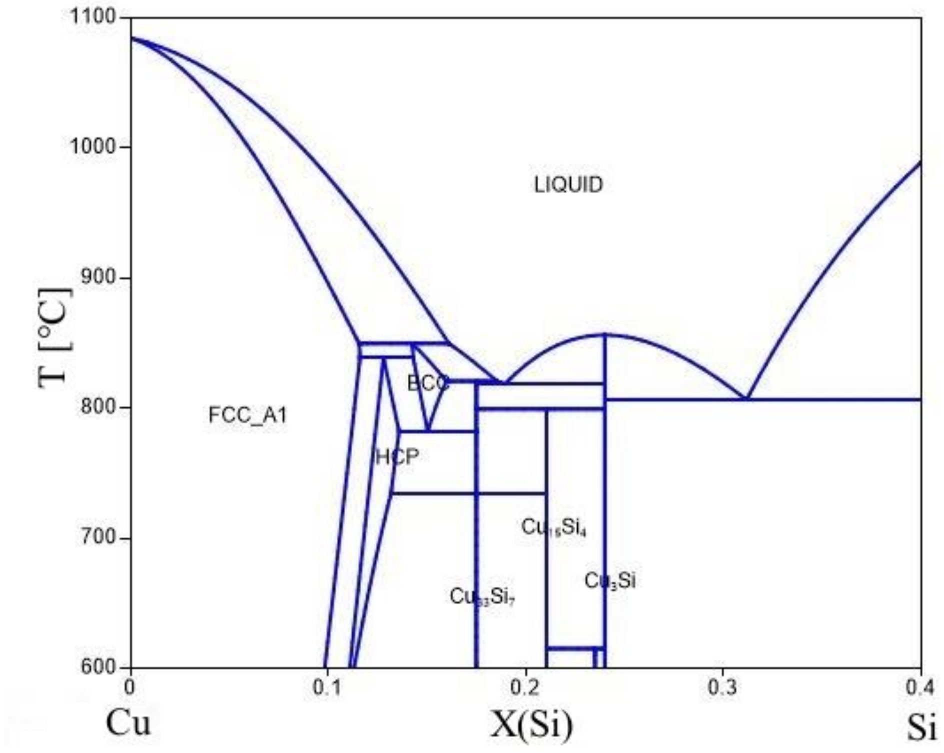

Figure S3. Cu-Si phase diagram obtained from the results of Hallstedt [22].

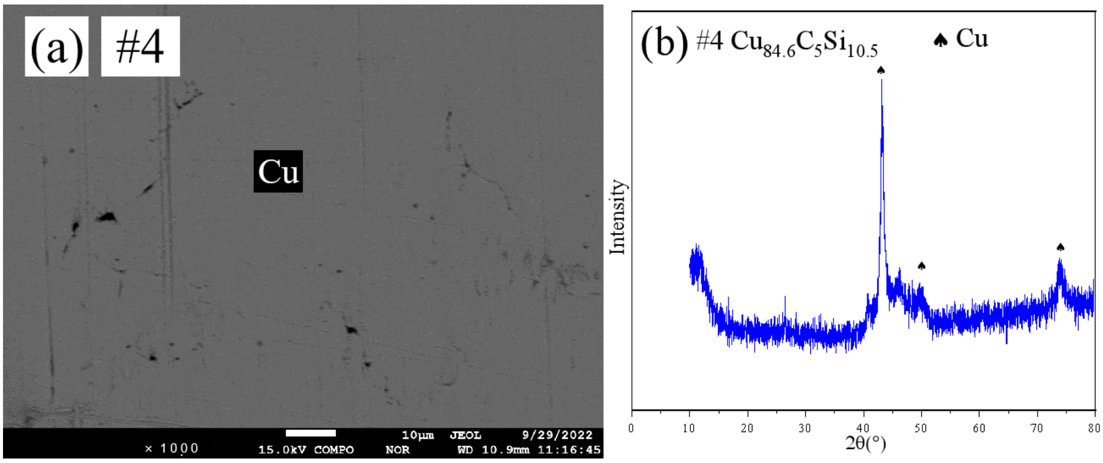

Figure S4. SEM and XRD patterns of sample 4: (a) SEM; (b) XRD.

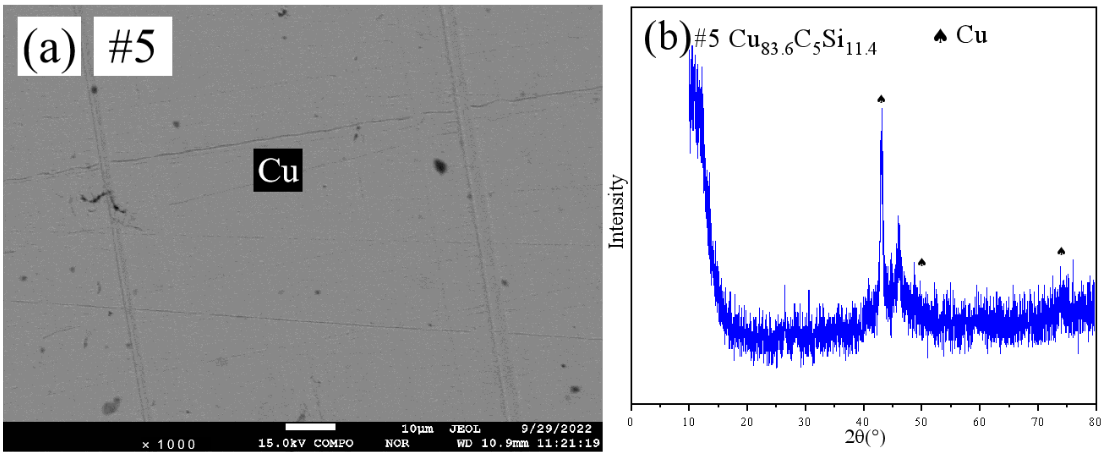

Figure S5. SEM and XRD patterns of sample 5: (a) SEM; (b) XRD.
